# Medical AI and AI for Medical Sciences

**DOI:** 10.31662/jmaj.2024-0185

**Published:** 2024-11-25

**Authors:** Kazuhiro Sakurada, Tetsuo Ishikawa, Junna Oba, Masahiro Kuno, Yuji Okano, Tomomi Sakamaki, Tomohiro Tamura

**Affiliations:** 1 Department of Extended Intelligence for Medicine, The Ishii-Ishibashi Laboratory, Keio University School of Medicine Graduate School of Medicine, Tokyo, Japan; 2 Department of Obstetrics and Gynecology, Keio University School of Medicine Graduate School of Medicine, Tokyo, Japan

**Keywords:** Artificial intelligence, Medical AI, AI for Medical Science, Machine learning, Deep learning, Large language model, P4 medicine

## Abstract

Digital transformation of healthcare is rapidly progressing. Digital transformation improves the quality of services and access to health information for users, reduces the workload and associated costs for healthcare providers, and supports clinical decision-making. Data and artificial intelligence (AI) play a key role in this process. The AI used for this purpose is called medical AI. Medical AI is currently undergoing a shift from task-specific to general-purpose models. Large language models have the potential to systematize existing medical knowledge in a standardized way.

The usage of AI in medicine is not limited to digital transformation; it plays a pivotal role in fundamentally changing the state of medical science. This approach, known as “AI for Medical Science,” focuses on pioneering a form of medical science that predicts the onset and progression of disease based on the underlying causes of disease. The key to such predictive medicine is the concept of “states,” which can be sought through machine learning. Using states instead of symptoms not only dramatically improves the accuracy of identification (diagnosis) and prediction (prognosis) but also potentially pioneers P4 medicine by integrating it with empirical knowledge and theories based on natural principles.

## Introduction

**Figure 1.**
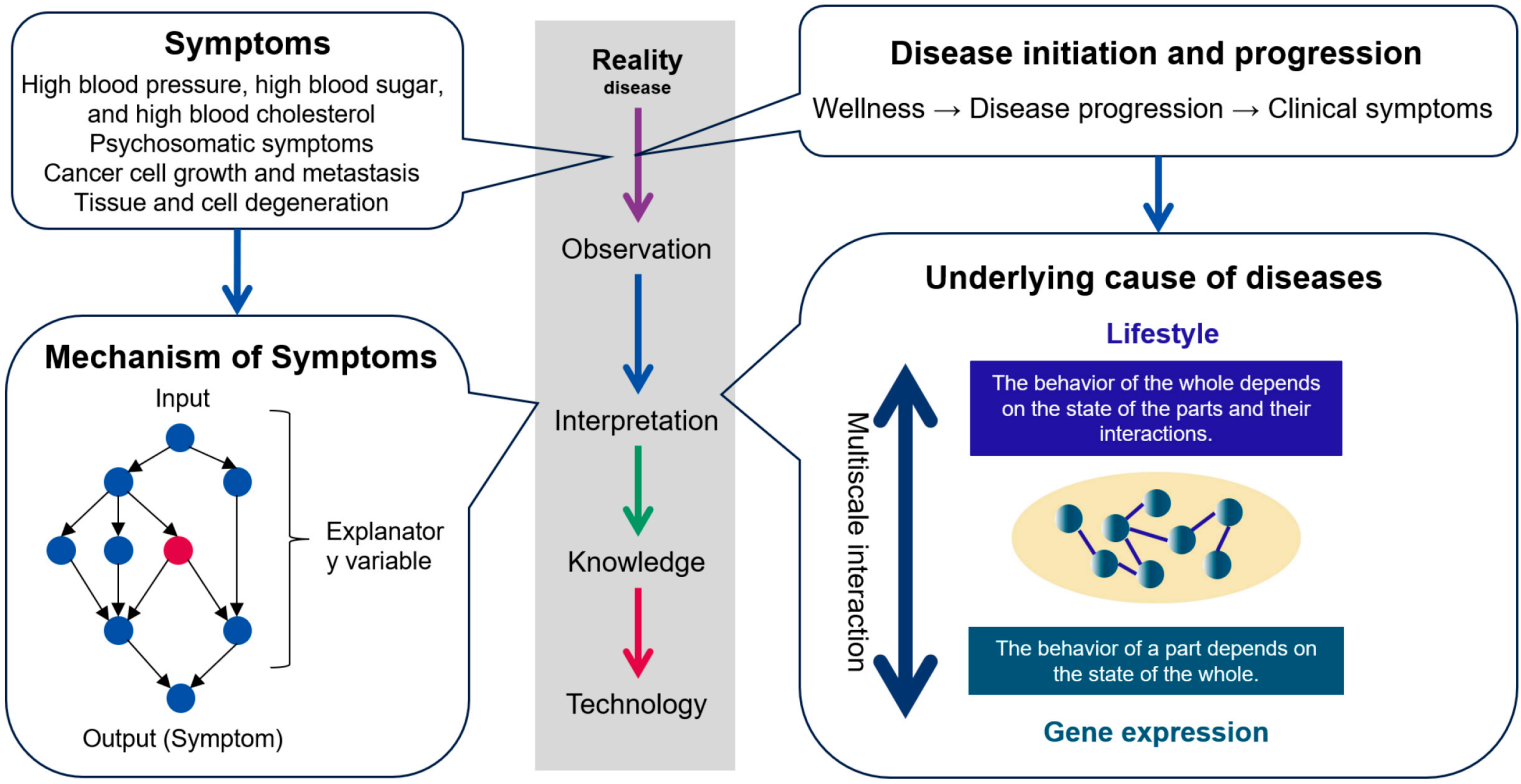
Two concepts of medicine Science is the process of observing and interpreting reality to gain knowledge. This knowledge is then applied to technology. In the past, medicine has focused on the symptoms of disease, interpreted them based on mechanisms, and found many causes for the symptoms. In the future, medicine must observe and interpret the process by which signs of disease appear, disease progresses, and symptoms appear. At this point, mechanism-based interpretation cannot be used. This is because disease emerges through a bidirectional dynamic process in which the global order emerges bottom-up through local interactions among system components and between components and the environment, and the global order thus created governs the local interactions among components top-down as a boundary condition. By understanding this emergent process, it is possible to get to the underlying cause of disease.

Maintaining the current healthcare system requires an exponential increase in healthcare costs, far exceeding the growth rate of gross domestic product (GDP) ^(1)^. This problem is exacerbated by an aging society and a declining population with declining birth rates as seen in Japan and many other countries. The incidence of chronic diseases, having a high prevalence in many countries, increases exponentially with age ^(2)^. New medical approaches that can prevent the onset of chronic diseases are required. This requires medical care that predicts and prevents the onset and progression of disease based on the underlying cause of the disease ^(3)^. The underlying causes of disease are the factors that define the transition to disease. Disease initiation and progression are determined by a complex of factors that include not only the genome but also various environmental factors and life stages. Future medicine will be based on two pillars: medicine that treats symptoms and medicine that treats underlying causes (Figure 1).

The technology of machine learning (ML), wherein computers automatically learn data and discover rules and patterns behind the data, is making great strides ^(4)^. Deep learning, the foundation of current artificial intelligence (AI), is a layered version of a type of ML called neural networks ^(5)^. In the medical field, AI models can automatically diagnose diseases or predict prognosis based on large amounts of clinical data used for training ^(6)^.

Innovations in medical science are required to achieve preventive medicine based on personalized and highly accurate predictions. For a long time, diagnoses were made using symptoms as indicators of disease, and treatments were implemented to alleviate symptoms. Accordingly, medical science has analyzed the mechanisms of symptoms, and drug research has developed treatments to counteract these mechanisms. However, unless measures are taken to address the underlying causes of disease, preventing the onset of chronic diseases will be impossible. Symptom-relieving medications only delay organ failure if the underlying causes of the disease are not treated. Medical care that individually predicts and prevents the onset of disease based on the underlying causes of disease is essential to the realization of a sustainable healthcare system. P4 medicine is a form of medicine that makes predictions and provides preventive, participatory treatment in a personalized way. A new scientific framework is required to build this new medical paradigm. To get to the underlying causes of disease, the new medical science should integrate three key elements: biological systems theory to comprehend the nature of disease systemically, deep phenotyping that traces the path from health to disease onset over time, and domain knowledge that explains diseases in terms of causality. Using AI to integrate these three and achieve the transformation of medicine represented by P4 medicine will be called “AI for Medical Science.”

## Overview of ML and Deep Learning in Medicine

**Figure 2.**
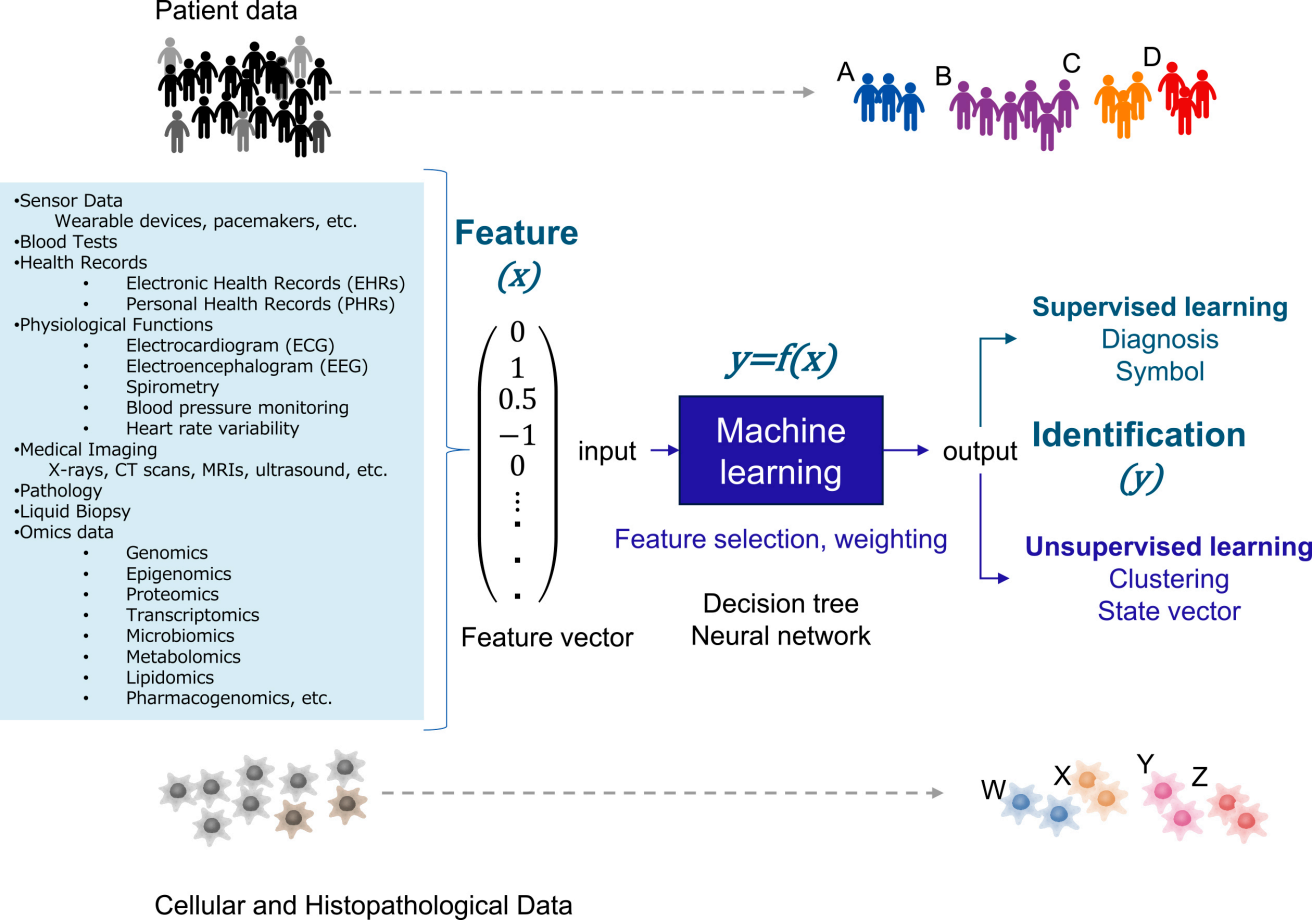
Analysis flow of ML Feature vectors are obtained from a large number of test data representing patient characteristics. At this point, feature engineering is performed. ML uses the feature vector as input (x) to produce the output (y). Supervised learning aims to reproduce predetermined outputs, whereas unsupervised learning extracts a hidden pattern from the feature vector.

ML is a branch of computer science to create predictive models (statistical models) from data. The data can be material, complex manmade systems (markets, factories, transportation, etc.) or complex natural systems (ecosystems, organisms, diseases, etc.). Predictive models are trained by algorithms on the data set. Models are represented by mathematical relationships between probabilistic and nonprobabilistic variables (Figure 2). In medicine, these learning models can be used for diagnosis and prognosis, given that sufficient reproducibility is guaranteed ^(6)^. Although biostatistics, clinical statistics, and genetic statistics usually analyze data hypothesizing certain mechanisms of a phenomenon, ML discovers the rules and patterns behind the data without assuming a mechanism.

Scalars, vectors, matrices, and tensors are used for data analysis. Scalar is a variable comprising only one number, such as blood glucose, blood pressure, or C-reactive protein. Vector is a variable comprising multiple scalars. Time and space information can be added using matrices and tensors when the state of the object is represented as a vector. Because ML extracts hidden patterns from patient clinical data, e.g., vital signs, laboratory data, and medical imaging data, comprising many variables, patient characteristics are represented as high-dimensional vectors (Figure 2). In ML, feature engineering is used to convert original data into feature vectors ^(7)^. Feature engineering comprises selecting the necessary variables from the clinical data and normalizing them. In normalization, the raw data values are converted to other values based on some index. For example, a Z-score is used to standardize the data to have a mean of 0 and a standard deviation of 1.

An ML model can be interpreted as a function to produce a desired output (y) from an input feature vector (x). ML algorithms for obtaining this function are neural networks, support vector machines, kernel methods, decision trees, ensemble learning, and other algorithms ^(6), (7)^. ML includes supervised learning, aiming to reproduce the output given in the training data, and unsupervised learning, identifying patterns and structures of the data based on feature similarity and regularity.

Deep learning is a type of an ML performed using multilayer neural networks ^(5)^. Unlike other ML algorithms, the features of the data are automatically found as the data are processed at each layer. Deep learning is effective for complex tasks such as object recognition in clinical images, speech recognition, and translation, where it is difficult for humans to explicitly design features.

Deep learning has used different network architectures for different tasks. A deep neural network is used for tasks that have no structure, such as tabular data or coordinates, a convolutional neural network for image recognition, a recurrent neural network for natural language, a generative adversarial network for image generation, and an autoencoder for dimensional reduction of input data ^(5)^. This structure was designed by humans to allow the network to adequately learn the characteristics of the data used for analysis. By contrast, the recently developed network architecture known as transformers can be applied to various tasks and achieves higher accuracy than conventional network architectures.

## Overview of Large Language Models

The use of generative AI based on large language models (LLMs; a type of foundation model), such as Generative Pretrained Transformer 4 (GPT-4), is expanding in the medical field ^(8)^. GPT has the ability to take multimodal input data such as text, images, and audio and compute an appropriate distribution as an output ^(9), (10)^. Text comprises words, images of pixels, and audio of amplitude and frequency. These elements are called tokens and are used to identify input variables. A token is a vectorized segment of a word, segmented image fragment, or segmented audio fragment. In addition, the position of these fragments is leveraged as features using positional encoding, as their arrangement of the fragments is crucial information. In this way, input data are encoded into a sequence of high-dimensional vectors. The four main functions of the transformer are self-attention, multilayer perceptron (MLP) block, in-context learning, and reinforcement learning from human feedback (RLHF) ^(11)^.

Self-attention has the ability to dynamically change the flow of data and can be used to learn rules about which information to attend to. Referring to related tokens at distant locations without losing information is also possible. In this case, the values (vectors) of individual tokens are influenced by the vectors of surrounding tokens of interest. For example, in text analysis, self-attention has the ability to select the necessary information from preceding past word sequences to predict the next word to appear. In this case, information from a distant word can be accurately captured.

The MLP block has the ability to determine which information to store and which information to associate with which information. Self-attention and the architecture of the MLP block make it possible to store all information in a web or book in a learnable format.

In-context learning is the ability to independently learn how to answer subsequent questions by simply adding a few examples to the input. This is a capability that LLMs have gained by learning how to learn themselves as they learn tasks. In-context learning makes it possible to perform entirely new tasks that have never been explicitly trained.

Goal-driven learning involves RLHF to improve the generated dialog. This process is done not only through human feedback but also with the supervision of the Instructor GPT.

## General-purpose Medical AI

Selecting the best test items for each patient and clinical reasoning for a wide variety of diseases depends on the medical knowledge of the physician. It is not easy for a single physician to master the ever-growing body of medical knowledge to diagnose and treat patients. AI that has absorbed a large amount of medical knowledge could be an ideal solution, allowing clinicians to use it to make decisions. The application of AI in this way is called medical AI.

More than 500 diagnostic devices with medical AI that have been cleared by the US Food and Drug Administration have been approved for only a few tasks ^(12)^. They were developed using ML and deep learning. Their models are task-specific and have been applied to chest X-rays, eye fundus images, cancer pathology images, electrocardiograms, echocardiograms, and other diagnostic procedures ^(6)^. Medical devices using the latest LLM have not yet been developed. Given the complexity of medical knowledge, it is difficult for such task-specific medical AI models to adequately support clinical practice.

**Figure 3.**
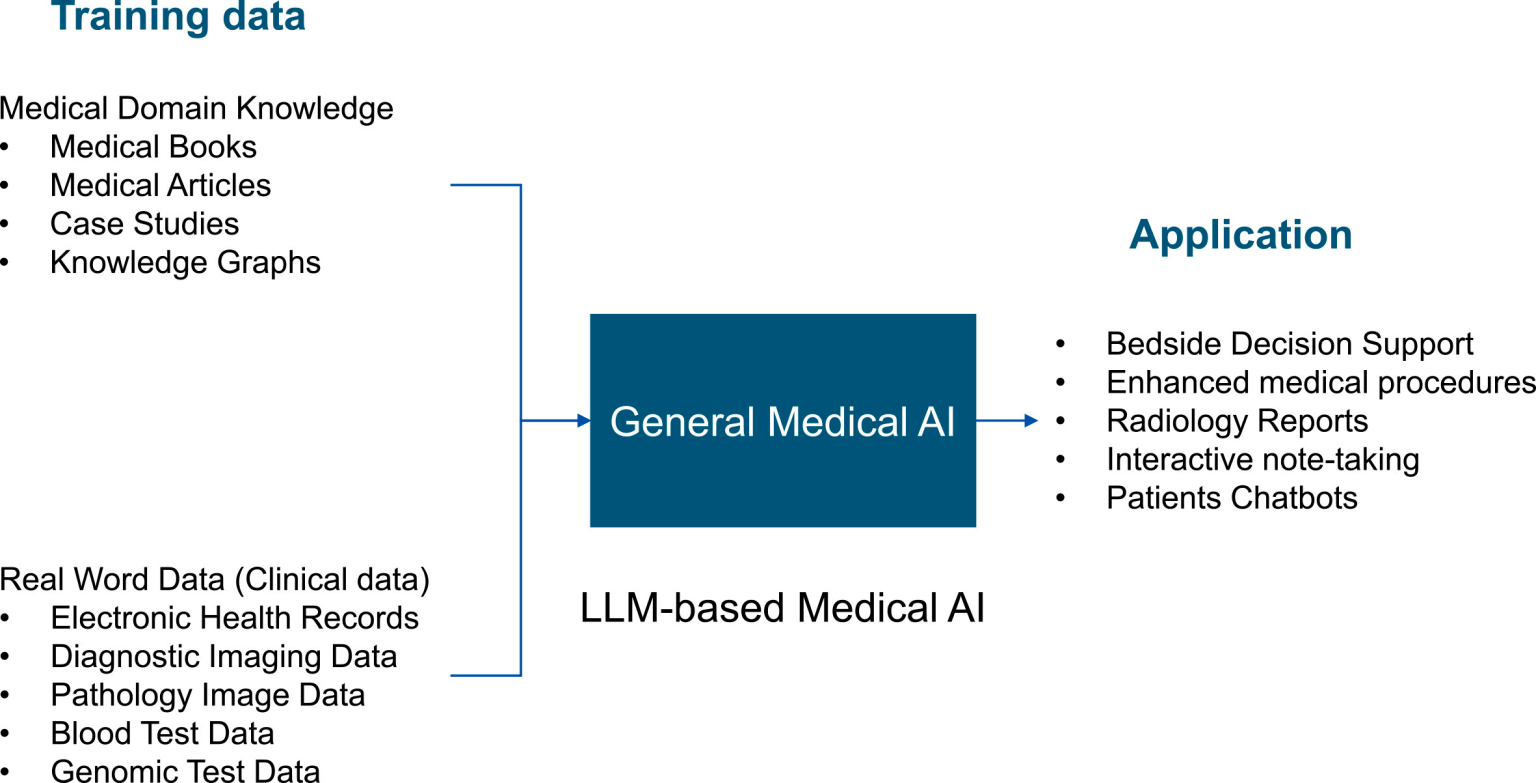
Development flow of a general-purpose medical AI model Consider the case of developing a general-purpose medical AI model using an LLM. In this case, medical domain knowledge and clinical data are used as training data. The general-purpose medical AI produced in this way can be applied to bedside decision support, medical procedure augmentation, radiology reporting, interactive note taking, and patient chatbots.

Inspired by LLMs, Moor et al. proposed a general-purpose medical AI model ^(13)^. Human knowledge, such as medical knowledge, comprises two main components: identification based on resemblance and causality ^(14)^. By combining image and examination data with textual information, all existing medical knowledge can be trained into an LLM (Figure 3). This standardization of medical knowledge through text and image is called a medical foundation model ^(13)^.

Medical foundation models can be applied to bedside decision support, digital radiology assistants, surgical assistance, automated documentation, patient-facing chatbots, and many other applications ^(13)^. In addition, a medical foundation model could solve even new clinical tasks that have never been trained on when given a task via text. To actually use medical foundation models in clinical settings, it is necessary to evaluate the reliability of the models and the reproducibility of the results. However, there are currently no quantitative indicators for this purpose. Once the specifications for the Medical Use Instructor GPT are finalized, it will be possible to automate the RLHF. This may prevent the phenomenon of the basic medical model generating false information (hallucinations). For example, if there are multiple conflicting answers in the learning model, or if there are no answers in the learning model, the generative AI must have the ability to mention this in the answers.

## Science and AI

**Figure 4.**
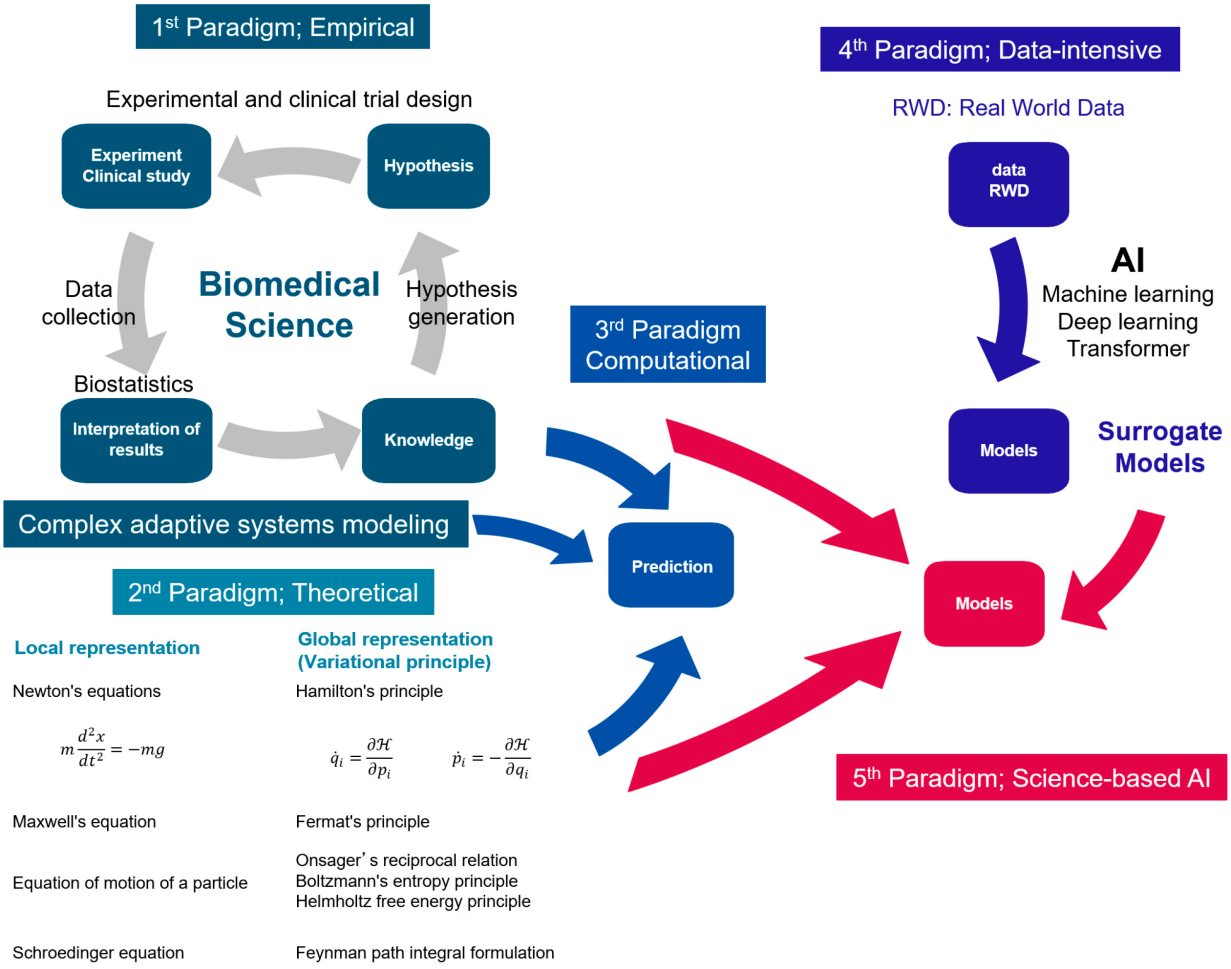
Scientific advances Science was pioneered by empirical science (first generation), represented by medicine and biology, and theoretical science (second generation), represented by physics and chemistry. These two paradigms were applied to computational science (third generation) as the capabilities of computers improved. By contrast, the progress of digital transformation has led to the collection and integration of large amounts of real-world data. Data-intensive science (fourth generation), which uses AI and ML to analyze this data, was pioneered. Today, science-based AI (fifth generation) is being developed, which incorporates knowledge from empirical science and principles from theoretical science into the surrogate models developed in data-intensive science.

Scientific discovery has evolved through four paradigms: empirical, theoretical, computational, and data-intensive (Figure 4) ^(15)^. Knowledge in the medical and biological sciences has been accumulated by testing causal hypotheses about disease through experiments and clinical trials. This is known as empirical science. Knowledge of causal mechanisms has been translated into mathematical models to approximate phenomena from governing equations. This interpretation of phenomena by simulation on a computer using mathematical models is called computational science. Medicine and biology do not have theories like physics.

In physics and chemistry, phenomena are interpreted based on natural laws. This is called theoretical science. Local representation using differential equations and global representation using the variational principle are two ways to understand natural laws. In physics, a theory in the form of differential equations was first established, followed by the establishment of a universal form of theory expressed in general coordinate systems, such as the variational principle.

Data-intensive science is modeled by analyzing real-world data with ML or deep learning. Models built from real-world data using ML or deep learning are called surrogate models because they are approximations of models based on scientific knowledge and natural principles. Recently, hybrid models have been developed, incorporating empirical and theoretical constraints into surrogate models, and they achieved high prediction accuracy. This approach is referred to as the fifth paradigm. ^(16)^.

The problem of predicting the three-dimensional structure of a protein from its amino acid sequence has so far been based on theories such as molecular dynamics calculations and quantum chemical calculations. In contrast, AlphaFold 2 achieves atomic-level structure prediction using a hybrid model that incorporates physical constraints into an ML model ^(17)^. AlphaFold 3, which takes into account intermolecular interactions, has also been developed ^(18)^.

Hypothesis-driven science comprises multiple steps: hypothesis generation; experimental design to test these hypotheses; data collection; and analysis. There is potential to innovate science by incorporating AI into each step of this process. This approach is called “AI for science” ^(19)^.

In the case of diseases that occur in the complex system of an organism, it is not possible to formulate appropriate governing equations because not all causal relationships are clear. Living organisms are open systems that are nonlinear and nonequilibrium. Approximating this with a linear model of causal mechanisms is only appropriate under limited conditions. The reason that diagnostics developed using ML are more predictive than multivariate analyses based on traditional biostatistics is that the algorithms are designed so that ML model such as random forest can capture the nonlinearity between variables. Medical discoveries, likewise in other scientific fields, are made through the multistep process of hypothesis-driven science introduced above. However, unlike other scientific fields, the concept of “AI for science” cannot be directly applied to medicine. This is because medicine does not involve natural principles in the way that physics does, and thus lacks the second paradigm.

## AI for Medical Science

Medicine comprises diagnoses based on symptom similarity and therapeutic interventions based on symptom mechanisms. Causal effects of mechanisms are tested by randomized controlled trials and examined by biostatistical analyses. Causality is established for a specific subject. Thus, the selection of subjects in comparable conditions is essential for reproducible causal estimation.

It is inevitable that medical science and medical care rely on symptoms, since symptoms are what we can observe about the phenomenon of disease. Nevertheless, there is diversity among patients exhibiting the same symptoms and labeled accordingly with the same disease. This diversity can alter the behavior of causal mechanisms that hold in stereotypical cases, resulting in diverse outcomes such as adverse drug events or nonresponse. In other words, populations that are symptomatically equivalent are not equivalent from different angles. This diversity may be related to differences in disease pathogenesis.

The onset and progression of disease are determined by a complex of factors, including not only the genome but also various environmental factors and life stages. Even diseases with the same symptoms can have different pathogenesis. However, we know little about the changes that occur in the early stages of disease onset because we have not been able to observe them. It is necessary to regularly measure the characteristics of the physical condition from the time of health in order to understand the causes of these changes that occur in the early stages of the disease. Clustering—or deep phenotyping—data obtained over time from a large number of samples using ML can visualize the onset and progression of disease that cannot be captured by symptoms.

Diagnosis is the process of identifying a patient based on similarities. The process of identifying a patient based on symptoms using natural language is called type. In genetics, the observable characteristics or traits of an organism are called phenotypes. In contrast, the process of identifying a patient based on numerical variables, such as clinical test values, is called state. When a patient is identified based on five clinical variables, the patient’s state is expressed as a point in a five-dimensional space. The clinical variables used in this case are called state variables. The abstract space wherein the states are arranged is called the state space. As the patient’s clinical variables change due to disease progression or treatment, the changes in the patient’s state are expressed as movement within the state space. This movement is fundamentally different from the movement of objects, so the term “time evolution” is generally used instead of “movement.” State variables are the smallest subset of system variables that can represent the state of the entire system at any given time. ML clustering is used to obtain state variables. Clustering is the representation of the set of states that the system can take in the state space.

But which features should be accounted and how should they be clustered? Or what does appropriate clustering mean? Key to this consideration lies in the definition of state. Living organisms are nonequilibrium, nonlinear, open systems in transition between different nonequilibrium steady states. To ground inference in reality, it is important to couple clusters to these nonequilibrium steady states. In this respect, unsupervised learning transforms feature vectors into state vectors so that similar states are located close together in the embedding space.

**Figure 5.**
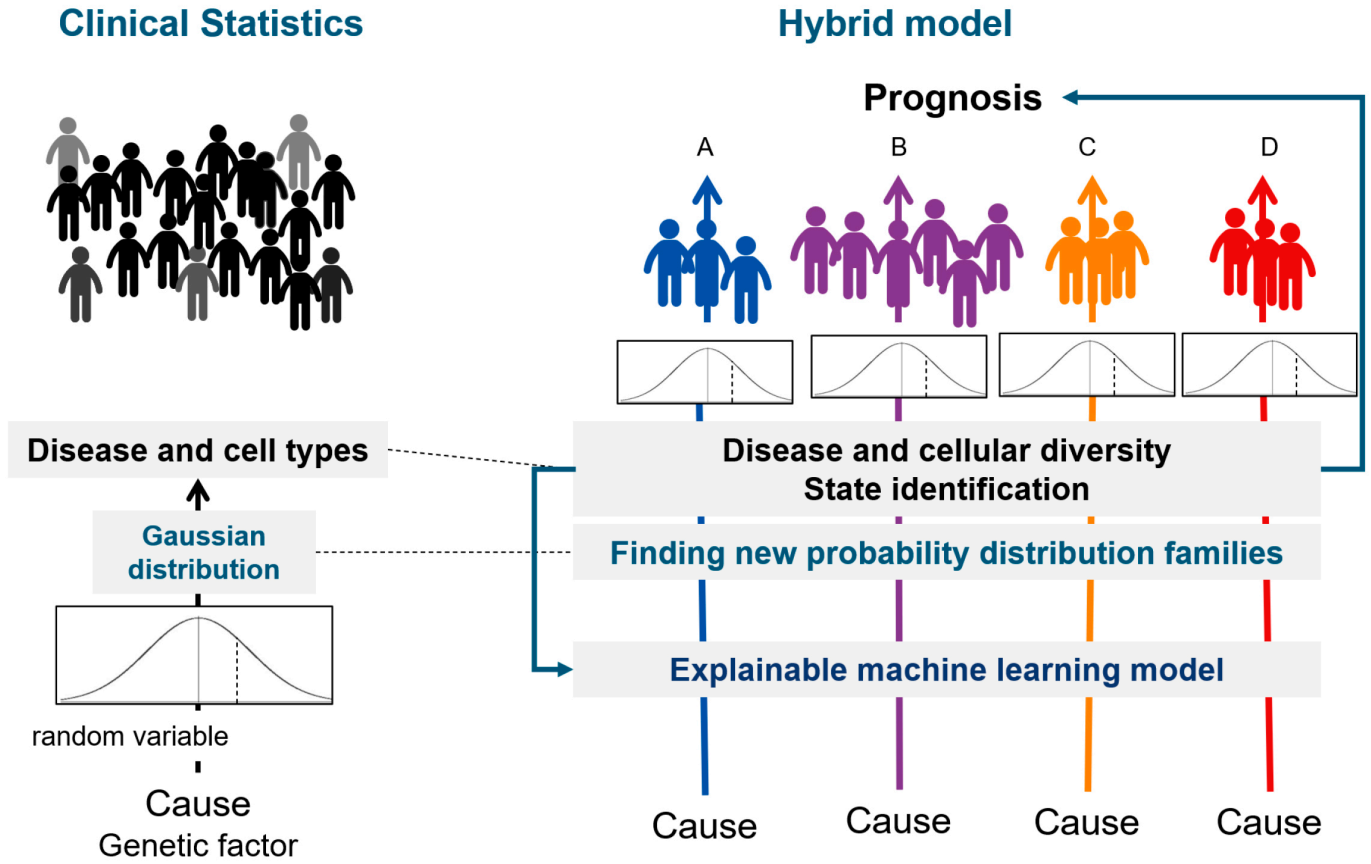
Fusion of causal inference and AI/ML In medicine, a group of patients with the same symptoms is considered a homogeneous Gaussian population, and the hypothesis of a causal mechanism is tested by clinical statistics. However, patients with the same symptoms often exhibit diversity in other characteristics. If this diversity is not taken into account in hypothesis testing, the causal hypothesis may not be adequately reproduced. To solve this problem, it is effective to use ML to classify patients with the same symptoms into clusters and then test the causal mechanism. This is called “AI for Medical Science.”

With appropriate clustering, any biological change can be represented as a transition from one state to another. Furthermore, the cause of this state transition can be identified using hypothesis testing (Figure 5). We call the inference that integrates biostatistics and ML “AI for Medical Science.”

## AI for Predictive Medical Science

**Figure 6.**
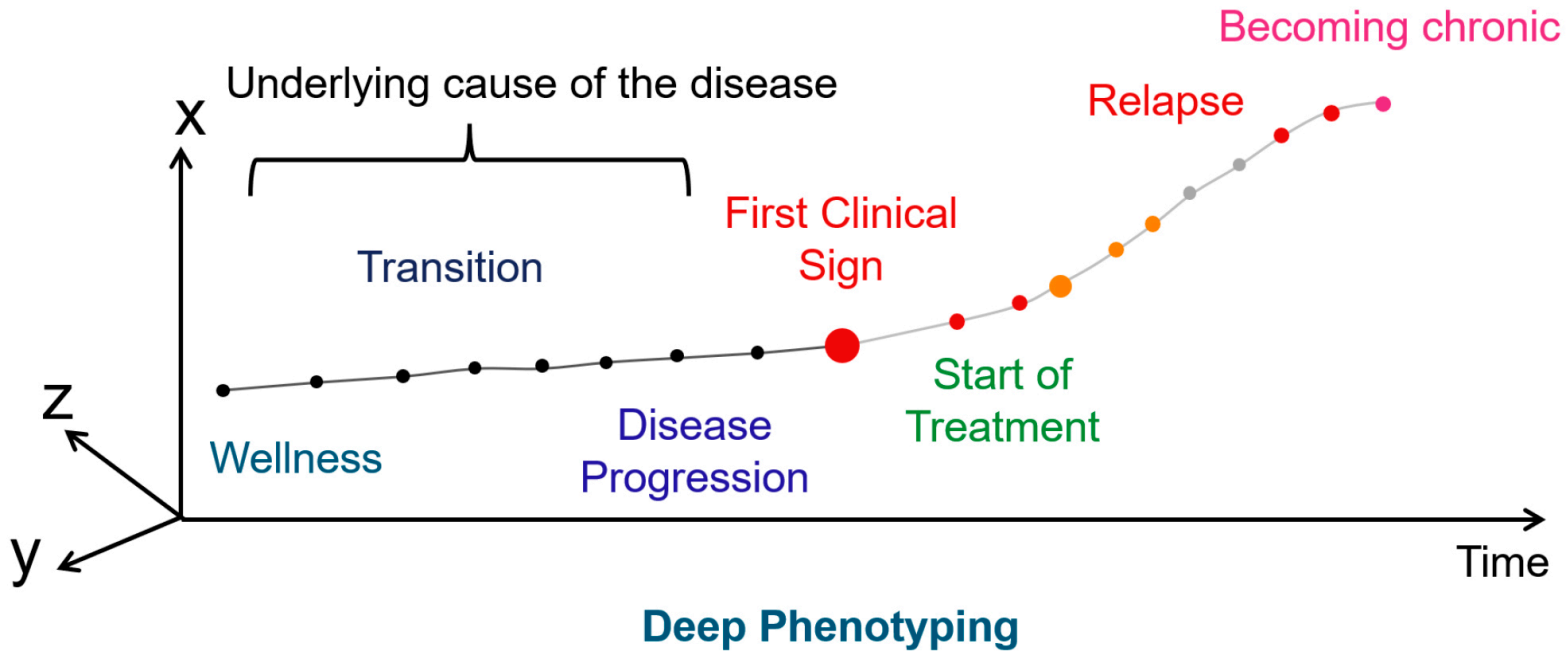
Standardization for life course inference A quantitative representation of the process by which a person undergoes disease progression as gradual transitions of physical states can numerically express clinical symptoms, recovery, or chronic phase after treatment. This approach is essential to realize preventive medicine based on highly accurate prediction. To achieve this, it is effective to represent a person as a state rather than a symptom and to represent state changes as trajectories in a state space.

From the moment of fertilization, humans go through development, growth, aging, and death, changing tissues, organs, and personal states. There are different states during this life course (Figure 6). Transitions from one state to another can be expressed in terms of causality and conditional probability. Conditional probability here means that given a state, the next transition to any state is expressed by probability.

Since a life course can be viewed as an array of tokens representing states ^(20)^, it becomes possible to apply inference to LLMs. LLMs for natural language systems predict the next word based on the previous text. Similarly, LLMs for life courses predict the next state from an array of previous state transitions. Transformers make it possible to search for the state required to predict the next state from the preceding sequence of past states. A large-scale disease model (LDM) can be developed by modeling all diseases in this way. LDM systematizes diseases based on physical states, unlike the infrastructure model that systematizes medical knowledge based on natural language. Such a model is called a physical state foundation model.

The basis for the realization of predictive medicine is to obtain a set of states that can be taken at each level of the human body, organs, tissues, and cells and to consider the life course as an array of states, with each state obtained here as a token.

## Physics-based Biomedical Science

The current framework of biology and medicine was agreed upon at the Princeton Conference on Genetics, Paleontology, and Evolution in 1947. First, genetics (functional biology) and evolutionary biology were integrated, followed by developmental biology in the 1970s ^(21)^. Two principals remaining effective were agreed upon: first, physics should not be involved in explaining life phenomena; and second, causal mechanisms should be used to explain life phenomena.

In addition to the machine analogy, life phenomena are explained with the help of information theory, which has emerged from information and communication technology. The complex adaptive system is adopted, in which the organism learns its own rules of behavior through interactions with its environment. Carl Friston proposed a concept called the free energy minimization principle wherein organisms are viewed as systems that predict environmental changes and learn to minimize prediction errors ^(22)^.

Diffusion models and GAN-based generative models can generate new images that do not exist before ^(23), (24)^. This technology has been applied to image generation AI services such as stable diffusion and Dall-E. It has also been shown that novel proteins with desired functions can be designed using foundation models of protein structure and function ^(25), (26)^. Thus, generative AI can be used to design new images and proteins desired by humans.

Emergence cannot be described by causal models, information theory, or diffusion models. In evolution, new functions, characteristics, and behaviors of living organisms emerge through the two-way interaction of systems and their components^(27)^. The onset of disease, like evolution, is an emergent change. Emergence is different from learning or design, where the goal is specified in advance. Explanations and predictions of life phenomena require reasoning based on natural principles that can incorporate emergence, in addition to reasoning based on causal mechanisms, probability, and information theory. One candidate is the principles of physics.

Physics lies at the core of such natural principles because it studies the principles of motion of objects. In contrast, the central thesis of medicine and biology is the transition of states in life. To bridge the gap between physics and life science, in our previous work, we developed a method called “organism mechanics.” It explains changes in states of an organism using the principles of physics by considering changes in state as movements in a state space ^(28)^. Organism mechanics has shown that emergent order formation proceeds according to the principle of maximum entropy generation. Oscillatory phenomena are observed in various biological phenomena at different scales. By considering organisms as oscillators, the property of order in biological reactions is explained through the synchronization of oscillators, while biological diversity is shown to arise from the violation of synchronization. Introduction of the principles of physics allows the interpretation of biological phenomena to be grounded in a deductive paradigm.

**Figure 7.**
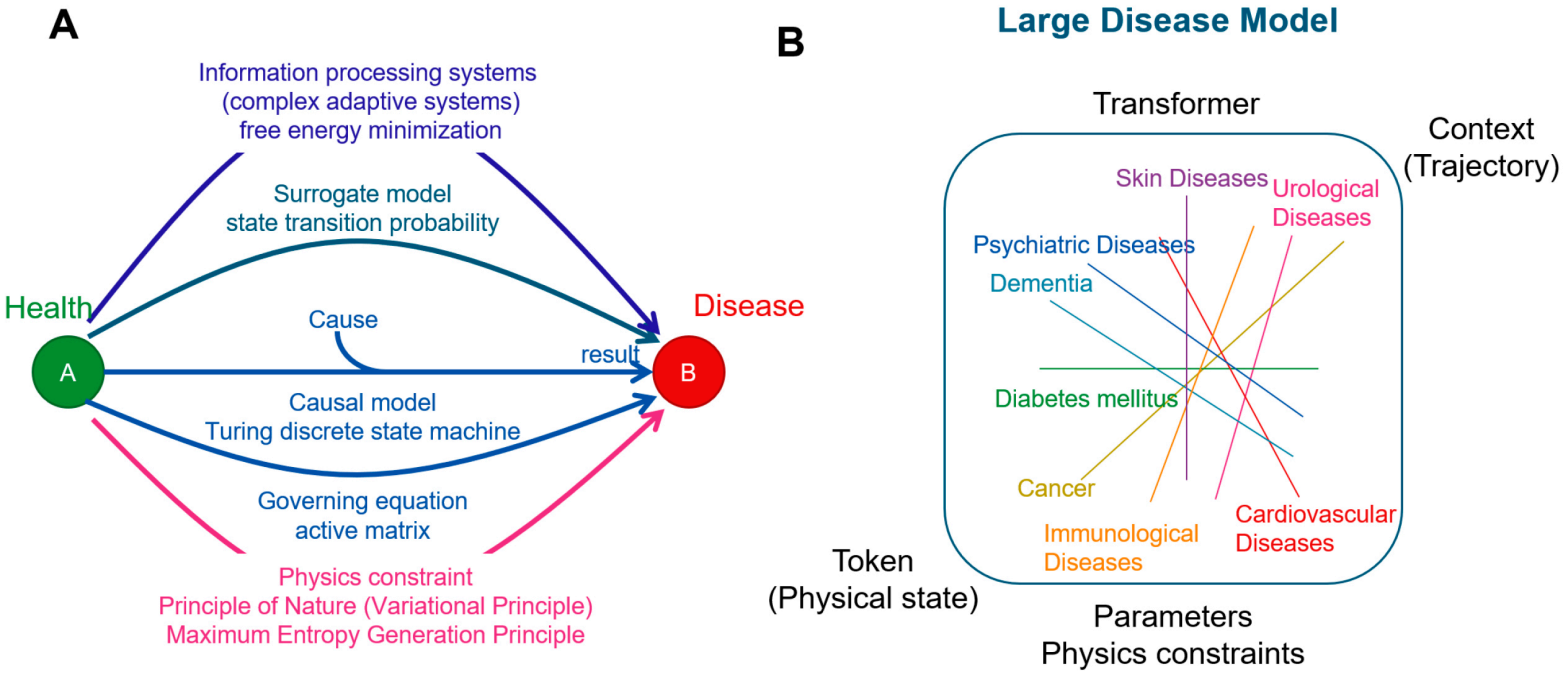
Large-scale disease model The challenge for medicine is to adequately predict and control changes in a person’s physical state. (A) There are five main ways to conceptualize such changes: (1) causal mechanisms; (2) simulation using governing equations; (3) probabilistic models using ML; (4) information theory; and (5) physical theory. To build a reproducible, reliable, and unified concept of diseases, it is necessary to develop a disease model that integrates these five lines of reasoning. (B) The final large language model of medicine will be a collection of all individual disease models that integrate the five inferences. Since all models share tokens (physical states) and contexts (state transitions), the models can be integrated using transformers. The reliability of the inferences will not only be parameterized based on data, but also physical principles will be introduced.

Life and ultimately disease phenomena can be explained and predicted by several different frameworks (Figure 7A). Disease models are developed by integrating different lines of reasoning. There are as many disease models as diseases themselves. We anticipate that large-scale disease model, an inclusive model of all disease models results (Figure 7B), will form the basis of future medicine, where causal, surrogate, and physics-oriented approaches are integrated harmoniously.

## Conclusion

Natural science, such as medicine, is about gaining knowledge by observing and interpreting reality. From this knowledge comes technology. A paradigm is the framework for observing and interpreting reality. A particular paradigm is shared within an academic field, and with the advancement of AI technology, a novel academic discipline is being established. This new perspective will lead to interpreting diseases in a different way, forming a groundbreaking architectonic of knowledge and next-generation medical technologies. In this study, we have introduced the idea of three new academic fields: AI for Medical Science; AI for predictive medical science; and organism mechanics.

## Glossary

### A) A medical foundation model

A large-scale artificial intelligence (AI) model that has been trained on a large amount of medical data using self- and semi-supervised learning and can be applied to various clinical tasks. The concept of a medical foundation model has been proposed, but it has not yet been developed.

### B) AI for Medical Science

It is necessary to standardize the identification of diseases in order to introduce the concept of AI for science into medicine. Identifying patients based on symptoms, such as diabetes and atopic dermatitis, is ambiguous in terms of disease differences. By clustering data using machine learning (ML), it is possible to identify patients based on their state. By combining this state identification with traditional biostatistics, it is possible to standardize medical reasoning.

### C) AI for predictive medicine

Medicine and biology are disciplines that explain phenomena that have occurred in the past, and it is not possible to predict what will happen in the future on an individual basis. To realize predictive medicine, we first need to express the patient’s physical state in a standard way. We can then develop a predictive model by expressing the change of states as a stochastic process, where the state at time *t* depends on the state(s) before time *t*. Machine learning can be used for state identification and predictive models. The inference performed in this framework is called AI for predictive medicine.

### D) AI for science

Scientific discovery is a multistep process that involves forming hypotheses, designing experiments to test those hypotheses, collecting data, and analyzing the data. AI for science is the innovation of scientific discovery by using AI to augment each step of this process.

### E) Artificial intelligence

AI is a computer system that has functions such as learning, reasoning, recognition, and judgment that are characteristics of human intelligence. In the second generation of AI, which began to be developed in the 1980s, the knowledge given to the computer had to be described by humans. In contrast, with the third generation of AI, which began to be developed in the 2000s, it became possible for AI to obtain knowledge from data itself through machine learning technology. Today’s AI is the third generation.

### F) Deep learning

This is a ML method that uses a multilayer neural network. It is possible to identify and predict by learning large amounts of data independently and automatically extracting features from the data.

### G) Diffusion models

The “diffusion model” is one of the generative AI models mainly used in AI services that generate image data. The mechanism of the diffusion model comprises two processes: the forward process, which adds Gaussian noise to the original image data; and the reverse process, which generates image data by denoising.

### H) Digital transformation

Digital transformation is a movement that aims to solve all kinds of problems, including business improvement, by making full use of digital technologies such as AI and big data.

### I) Foundation models

Foundation models are large-scale AI models that have been trained on large amounts of data using self- or semi-supervised learning and can be applied to various downstream tasks.

### J) GAN-based generative models

Generative adversarial networks (GAN) is a type of generative model that can generate data that does not exist by learning features from data. GAN is an “unsupervised learning” method that learns features without providing the correct data. GAN comprises two blocks: generator and discriminator. Both blocks use neural networks. The generator receives random numbers as input and outputs new data. The discriminator judges whether the input data is generated by the generator or a real one included in the training data. Thus, the relationship between the generator and discriminator is adversarial.

### K) In-context learning

In-context learning is a method of training a large language model by giving it examples of tasks as prompts. By simply giving it prompts, it will be able to perform various tasks without additional training.

### L) Large language models

Large language models (LLMs) are a type of foundation model that refers to models that have been self-trained on large amounts of text data. GPT-3 and GPT-4 are examples of LLMs.

### M) Machine learning

ML is a branch of computer science that deals with the construction of learning algorithms. Given input data, an algorithm is used to build a statistical model that produces the desired output. This input is called training data.

### N) Medical AI

Medical AI can independently perform tasks such as diagnosis, prognosis, treatment selection, and surgery based on existing medical knowledge. It supports doctors in a role similar to that of a co-pilot. The medical foundation model is a specific example of medical AI. The medical foundation model supports clinical decisions based on various clinical data. When a medical robot is developed, a medical AI is developed that makes clinical decisions based on data collected by the robot. This AI has different functions from the medical foundation model.

### O) Multilayer perceptron block

The attention outputs are projected, normalized, and then processed by a multilayer perceptron (MLP) block. This MLP block typically comprises two layers separated by a nonlinear activation function.

### P) Organism mechanics

Organism mechanics is a fundamental theory that describes the behavior of living systems using the principles of physics. The basic theory was developed by Sakurada and Ishikawa. Organisms create coordination at every level through information transfer. The state changes of organisms were formalized using the force of “information potential” and “information flow.” From this formalization, the basic principles of life were derived, including the principle of maximum entropy generation.

### Q) P4 medicine

This is a representation of how medicine will develop in the future, using the “4 Ps.” It was proposed by the American biologist Dr. Leroy Hood. The four Ps stand for personalization, prediction, prevention, and participation. “Personalization” means tailoring treatment to the constitution of each individual patient. Until now, the focus of medical treatment has been on responding to the symptoms of patients who have become ill. In contrast, the idea is that we should focus on “prevention” to make it harder to get sick by “predicting” the signs of illness. Participation means that it is important for patients to be proactive in their treatment and health maintenance.

### R) Reinforcement learning from human feedback

Goal-directed learning to enable large-scale language models to respond according to user intent. The model is modified using reinforcement learning. Specifically, a person, called a “labeler,” evaluates whether or not the response generated by the dialog system is appropriate by providing prompts. It is not efficient to leave all this feedback to humans, so we let the AI do the goal-directed learning. The AI with this function is Instructor GPT.

### S) Self-attention

The self-attention mechanism is a system that determines which parts of the data are important and which parts should be focused on when an AI is performing a task. The transformer is a network architecture that uses the self-attention mechanism as its core.

### T) Surrogate models

Surrogate modeling is a method of calculating and predicting phenomena using ML instead of numerical simulation. In medicine, it means using ML models to make diagnoses and predict prognoses instead of making inferences based on disease mechanisms.

### U) Transformer

A transformer is a network architecture that can be widely applied to various tasks. It processes data by alternately stacking units called self-attention mechanisms and MLP blocks.

## Article Information

### Conflicts of Interest

None

### Sources of Funding

This work was supported by Keio University Ishii/Ishibashi Foundation.

### Acknowledgement

We would like to thank the members of the Department of Extended Intelligence for Medicine at Keio University School of Medicine for their valuable insights and feedback.

### Author Contributions

K.S., writing - original draft, conceptualization, investigation, and visualization. T.I., J.O., M.K., Y.O., T.S., and T.T., writing - review and editing.
